# Worldwide Assessment of Low- and Middle-Income Countries' Regulatory Preparedness to Approve Medical Products During Public Health Emergencies

**DOI:** 10.3389/fmed.2021.722872

**Published:** 2021-08-13

**Authors:** Alireza Khadem Broojerdi, Claudia Alfonso, Razieh Ostad Ali Dehaghi, Mohamed Refaat, Hiiti Baran Sillo

**Affiliations:** Regulatory Systems Strengthening Team, Regulation and Safety Unit, World Health Organization, Geneva, Switzerland

**Keywords:** World Health Organization, regulatory preparedness, regulatory preparedness during public health emergencies, medical products approval, global benchmarking tool, regulatory systems strengthening, regulatory response, regulatory capacity building

## Abstract

**Background:** Regulatory preparedness for public health emergencies is critical. However, responses to past emergencies, such as the 2009 H1N1 influenza pandemic and medical product shortages, have revealed sizable gaps in countries' regulatory capacity and preparedness. A systematic analysis of the regulatory preparedness of countries around the world has not yet been performed. The purpose of this study was to analyze and document the current regulatory preparedness status, highlight the related gaps and challenges in order to propose strategic, harmonized, and sustainable regulatory solutions to improve future responses to public health emergencies.

**Methods:** From 2016 to 2020, we used the World Health Organization (WHO)'s Global Benchmarking Tool (GBT), a standardized instrument for identifying national regulatory authorities' strengths and gaps, to analyze the regulatory preparedness of 84 Member States, 95% of which were low- or middle-income countries. We analyzed whether participating Member States had not implemented, displayed ongoing implementation, had partially implemented, or had fully implemented 10 of the GBT's 268 sub-indicators most relevant to regulatory preparedness for public health emergencies.

**Findings:** Only 10 Member States (12%) that underwent benchmarking had fully implemented all 10 sub-indicators related to regulatory preparedness for public health emergencies; 34 (40%) had fully implemented ≥50% of the emergency sub-indicators, and 20 (24%) had not fully implemented any of the sub-indicators. With regard to individual sub-indicators, regulatory preparedness ranged from 19 Member States (23%) fully implementing reliance on clinical trial decisions of others to 45 (59%) fully implementing legal provisions to fast-track (or expedite) marketing authorization applications.

**Interpretation:** Many WHO Member States have limited regulatory preparedness for a public health emergency. Strengthening regulatory systems and promoting Good Regulatory Practices and reliance in these countries, to enable efficient response to emergencies, should be a global health priority.

## Research in Context

*Evidence before this study*. Responses to past public health emergencies have highlighted important gaps in regulatory preparedness around the world. For example, during the 2009 H1N1 influenza pandemic, limitations in streamlining national registration procedures contributed to a situation in which some countries were not able to promptly approve and use vaccines. Other emergencies have also revealed preparedness shortcomings that limited access to important medical products, such as the 2014 and current Ebola outbreaks, the switch in the vaccine used in the polio eradication initiative, and medical product shortages (to which markets in low-and middle-income countries are especially vulnerable). Although these findings indicate that the regulatory systems of many Member States are unprepared for public health emergencies, no systematic analysis of the regulatory preparedness of countries around the world has yet been performed.

*Added value of this study*. This study provides insight into the regulatory preparedness of 84 primarily low- and middle-income countries to respond to public health emergencies. It shows that many WHO Member States have limited regulatory preparedness to approve medical products during a public health emergency.

*Implications of the available evidence*. The findings of this study will help Member States, donors, and technical partners understand the current state of regulatory preparedness for public health emergencies around the world. By highlighting Member States' current regulatory preparedness in a variety of areas, such as registration and marketing authorization, vigilance, regulatory inspections, and clinical trials, the study will help target resources to where they are most needed. Finally, the results of this study emphasize that strengthening regulatory systems in low- and middle-income countries, to enable an efficient response to emergencies, should be a global health priority.

## Introduction

It is critical that every country has a regulatory system for medical products that is prepared to deal with public health emergencies. However, past responses have revealed gaps in emergency preparedness. The 2009 H1N1 influenza pandemic, for example, highlighted the need to shorten timelines for regulatory processes during emergencies, especially for vaccines. Limited streamlining of national registration procedures contributed to a situation in which some countries were not able to promptly approve and use influenza vaccines (1, 2). Other emergencies have also revealed preparedness shortcomings that limited access to important medical products, such as the 2014 and current Ebola outbreaks, the switch in the vaccine used in the polio eradication initiative, and medical product shortages (to which markets in low-and middle-income countries, or LMICs, are especially vulnerable) (3–6). Now, the COVID-19 pandemic presents an even bigger challenge: multiple medical products, new indications, new technologies, and an urgent need around the world.

Preparing for public health emergencies is a matter of human rights and health equity. Loss of lives in LMICs can be prevented through timely access to critical and life-saving products. Therefore, strengthening regulatory systems to enable an efficient response to emergencies in these countries should be a global health priority (7), and the World Health Organization (WHO) has an important role in helping Member States prepare for public health emergencies. In fact, one of the items in the WHO's 5-year regulatory action plan is to “strengthen preparedness for entry of medicines, vaccines, and other health products in countries experiencing a public health emergency or crisis” (8). In addition, one of the three strategic priorities in WHO's 13th General Program of Work is to leave “one billion more people better protected from health emergencies” (9).

WHO helps Member States strengthen their regulatory systems for medical products by setting norms and standards, promoting smart regulation, helping identify strengths and gaps, and providing specialized technical assistance, capacity building opportunities, and advice (10). The primary means by which WHO evaluates regulatory systems is through its Global Benchmarking Tool (GBT), a standardized instrument for identifying a national regulatory authority (NRA)'s strengths and gaps (10). In WHO's approach to capacity building, the GBT is used to benchmark national regulatory systems, leading to the formulation of institutional development plans (IDPs) aimed at sustaining NRAs' strengths and addressing their gaps. WHO provides technical support, as well as training, learning, and networking opportunities, to enable NRAs to implement their IDPs and monitor progress toward their goals (10).

The latest version of the GBT (revision VI), released in 2018, encompasses 268 sub-indicators in total, evaluating (1) registration and marketing authorization, (2) vigilance, (3) market surveillance and control, (4) licensing establishments, (5) regulatory inspection, (6) laboratory testing, (7) clinical trials oversight, and (8) national regulatory authority lot release, all under the overall umbrella of regulatory system functioning (10, 11). The purpose of the current study was to use data collected with the GBT to analyze the current regulatory preparedness status of Member States in all WHO regions, in order to propose strategic, harmonized, and sustainable regulatory solutions to improve future responses to public health emergencies.

## Methods

This study included all WHO Member States that underwent either self- or formal benchmarking using the GBT from 2016 to 2020. Many underwent benchmarking to strengthen their regulatory systems; benchmarking is step 2 of the WHO's 5-step approach to NRA capacity building (10). Others did so because only countries with NRAs that have achieved an overall maturity level of 3 or 4 are eligible to apply for WHO prequalification of the vaccines they produce. [After benchmarking, an NRA is assigned an overall maturity level from 1 (existence of some elements of regulatory system) to 4 (operating at advanced level of performance and continuous improvement); an NRA's overall maturity level is based on the lowest maturity level of any individual regulatory function (10)] Member States supplied GBT data with the understanding that no information that could be linked back to an individual country would be shared outside of WHO.

### Benchmarking Process

When a Member State undergoes benchmarking, each of the GBT's 268 sub-indicators is rated as not implemented, displaying ongoing implementation, partially implemented, or fully implemented (10).

#### Self-Benchmarking

First, Member States perform self-benchmarking, to identify strengths and areas for improvement. A computerized version of the GBT (cGBT) is used as a rubric to assess an NRA's maturity level. Staff from the relevant departments or units of an NRA self-score each GBT sub-indicator and provide evidence to justify their scoring, including links to publicly available information or files uploaded to the WHO NRA information-sharing platform. In the unfortunate case where sub-indicators are not scored by the respective NRA, data is shown as “not available.” The latest versions of the cGBT, cGBT manual and related procedures, and an online training module are available on the secure WHO Regulatory Systems Strengthening information-sharing platform. They can be accessed by participating NRAs or requested by other interested stakeholders. During the self-benchmarking process, NRA staff also have the opportunity to propose input to the initial IDP. WHO staff review responses and evidence to verify the information in the GBT self-benchmarking evaluation. WHO often assists Member States by organizing self-benchmarking workshops in which WHO staff explain how the GBT works, the importance of accurate assessment, and expected outcomes, as well as respond to questions.

#### Formal Benchmarking

Formal benchmarking takes place after the self-benchmarking and verification process has concluded. The goal of formal benchmarking is for a team of WHO assessors to evaluate the participating NRA by benchmarking its different regulatory functions, again using the cGBT as a rubric. A country's decision to undergo formal benchmarking typically includes consideration of its willingness to undergo the process, its objective (for example, to obtain eligibility to apply for WHO vaccine prequalification), and the availability of necessary resources, in addition to the satisfactory completion of self-benchmarking. At the conclusion of the formal benchmarking process, WHO generates a set of recommendations and formulates an IDP to address identified gaps. Following implementation of all critical recommendations, WHO issues an official letter indicating the maturity level of an NRA's overall system (GBT Maturity Level 3 being the target), as well as the maturity level of individual regulatory functions.

#### GBT Sub-indicators Relevant to Public Health Emergencies

Ten GBT sub-indicators are considered directly relevant to regulatory preparedness for public health emergencies (Table 1). Of the 10 sub-indicators, three evaluate an NRA's overall system-related activities, whereas seven evaluate individual regulatory functions (registration and marketing authorization, vigilance, regulatory inspection, and clinical trials oversight). All 10 sub-indicators were considered in this study.

**Table 1:** Global benchmarking tool sub-indicators relevant to emergency preparedness.

**Sub-indicator**	**Area/function evaluated**
**RS03.04:** Documented policies, procedures, and mechanisms, including written criteria, are established for recognition and reliance on decisions of other national regulatory authorities, or NRAs (if applicable).	National regulatory system
**RS04.05:** Written criteria to cover circumstances in which the routine regulatory processes may not be followed in relation to crises and emergencies linked to a risk management plan.	National regulatory system
**RS07.03:** There are provisions relating to reduction or exemption of dues, taxes, tariffs, or fees in defined situations for public health interest.	National regulatory system
**MA01.06:** There are legal provisions to cover circumstances under which the routine marketing authorization procedures may not be followed (e.g., for public-health interests).	Registration and marketing authorization
**MA01.08:** Legal provisions and/or regulations allow the NRA to recognize and/or rely on marketing authorization–relevant decisions, reports, or information from other NRAs or regional and international bodies.	Registration and marketing authorization
**MA01.12:** There are established guidelines that cover circumstances under which the routine marketing authorization procedures may not be followed (e.g., for public- health interest)	Registration and marketing authorization
**MA04.07:** There are documented mechanisms to handle non-routine registration and marketing authorization requirements in special situations (e.g., public-health interest).	Registration and marketing authorization
**VL04.06:** The NRA has access to expert committees for review of serious emergent safety concerns, when needed.	Vigilance
**RI01.05:** Legal provisions and regulations allow the recognition of and/or reliance on foreign NRA inspections and enforcement actions based on well-defined criteria.	Regulatory inspection
**CT01.11:** Legal provisions and/or regulations allow the NRA to recognize and use relevant clinical trial decisions, reports, or information from other NRAs, or from regional and international bodies.	Clinical trials

### Data Analysis

After benchmarking is performed, the results are stored securely in a WHO database that contains all regulatory systems strengthening information gathered since 1997 and allows limited users to quickly analyze data, stratifying by variables of interest. This database was used to complete the current study, which assessed the implementation level of each of the 10 public health emergency–related sub-indicators individually, as well as implementation of this group of sub-indicators as a whole.

## Results

### Characteristics of Participating Member States

**Figure 1:**
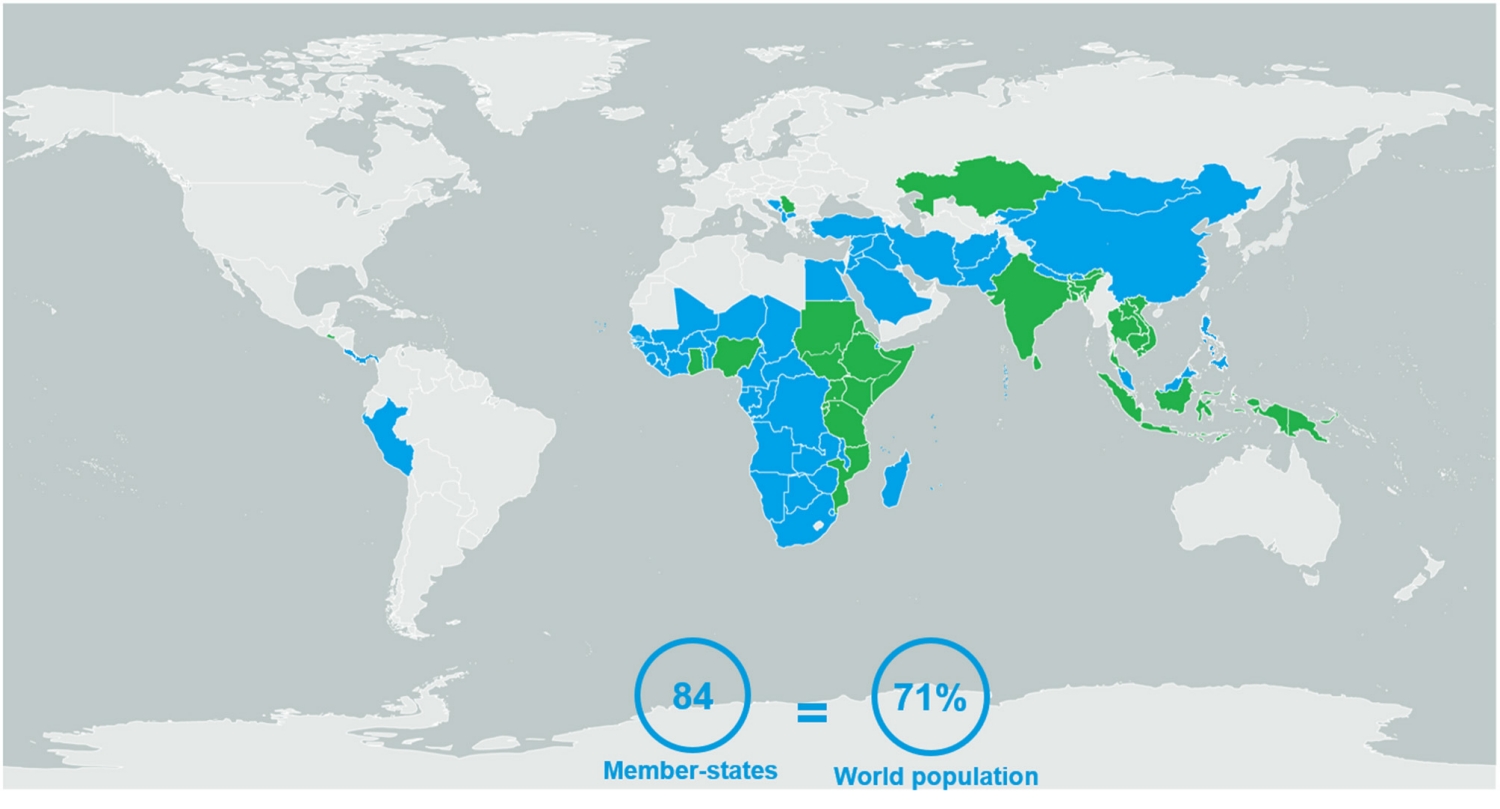
The 84 Member States evaluated using the WHO's Global Benchmarking Tool between 2016 and 2020, either *via* self- (*blue*) or formal (*green*) benchmarking.

This study included GBT results obtained from 84 of 194 Member States, consisting of 71% of the world population, according to World Bank data (Figure 1) (12). Benchmarking was performed from 2016 to 2020; 58 of the Member States (69%) underwent self-benchmarking and 26 (31%) underwent formal benchmarking (Table 2). Only four participating Member States (5%) were considered high-income, according to World Bank classifications (13); the rest were LMICs (Table 3). Most participating Member States were from Africa or Asia (Figure 1).

**Table 2:** The 84 member states evaluated using the WHO's global benchmarking tool between 2016 and 2020.

**Self-benchmarking**	**Formal benchmarking**
1. Afghanistan	1. Bangladesh
2. Albania	2. Burundi
3. Angola	3. Cambodia
4. Benin	4. El Salvador
5. Bhutan	5. Eritrea
6. Bosnia and Herzegovina	6. Ethiopia
7. Botswana	7. Ghana
8. Burkina Faso	8. India
9. Cameroon	9. Indonesia
10. Cabo Verde	10. Kazakhstan
11. Central African Republic	11. Kenya
12. Chad	12. Lao People's Democratic Republic
13. China	13. Mozambique
14. Comoros (the)	14. Nigeria
15. Congo	15. Papua New Guinea
16. Costa Rica	16. Rwanda
17. Côte d'Ivoire	17. Serbia
18. Democratic Republic of Congo	18. Somalia
19. Djibouti	19. South Sudan
20. Egypt	20. Sri Lanka
21. Equatorial Guinea	21. Sudan
22. Eswatini	22. Thailand
23. Gabon	23. Timor-Leste
24. Guinea	24. Uganda
25. Guinea-Bissau	25. United Republic of Tanzania
26. Iraq	26. Viet Nam
27. Iran (Islamic Republic of)	
28. Jordan	
29. Kyrgyzstan	
30. Lebanon	
31. Liberia	
32. Madagascar	
33. Malawi	
34. Malaysia	
35. Maldives	
36. Mali	
37. Mauritius	
38. Mongolia	
39. Montenegro	
40. Namibia	
41. Nepal	
42. Niger	
43. North Macedonia	
44. Pakistan	
45. Panama	
46. Peru	
47. Philippines	
48. Saudi Arabia	
49. Senegal	
50. Seychelles	
51. Sierra Leone	
52. South Africa	
53. Syrian Arab Republic	
54. The Islamic Republic of the Gambia	
55. Togo	
56. Turkey	
57. Zambia	
58. Zimbabwe	

**Table 3:** Characteristics of 84 member states evaluated using the WHO's global benchmarking tool (GBT), 2016–2020.

	**Countries benchmarked, *n* (%)**
**Benchmarking year**	
2016	9 (11)
2017	24 (29)
2018	11 (13)
2019	29 (35)
2020	11 (13)
**Type of benchmarking**	
Self	58 (69)
Formal	26 (31)
**Income level**	
Low	25 (30)
Lower-middle	32 (38)
Upper-middle	23 (27)
High	4 (5)
**WHO region**	
Africa	43 (47)
Americas	4 (5)
Eastern Mediterranean	12 (14)
European	8 (9)
South-East Asia	9 (11)
Western Pacific	8 (10)

### National Regulatory System Sub-indicators

**Figure 2:**
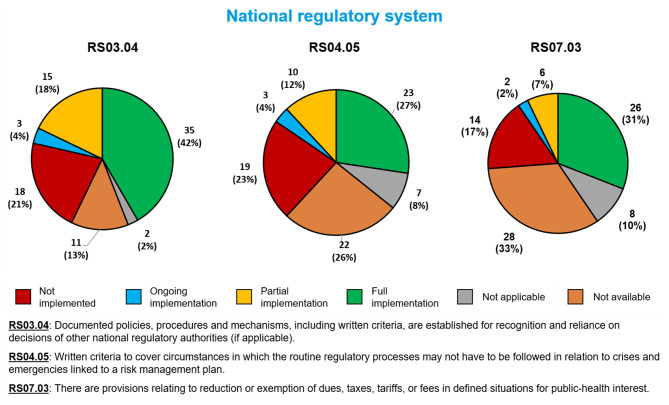
Implementation of three WHO Global Benchmarking Tool sub-indicators related to the overall functioning of a national regulatory authority in public health emergencies, among 84 Member States benchmarked from 2016 to 2020.

Fewer than half of Member States fully implemented each of the three sub-indicators related to the overall functioning of an NRA with regard to public health emergencies (Figure 2). The sub-indicator most often fully implemented involved reliance on the decisions of other NRAs (RS03.04, 35 Member States, 42%). The sub-indicator least often fully implemented involved special responses to public health emergencies (R04.05, 23 Member States, 27%). Twenty-six Member States (31%) had fully implemented fee exemption during public health emergencies (RS07.03).

### Registration and Marketing Authorization Sub-indicators

**Figure 3:**
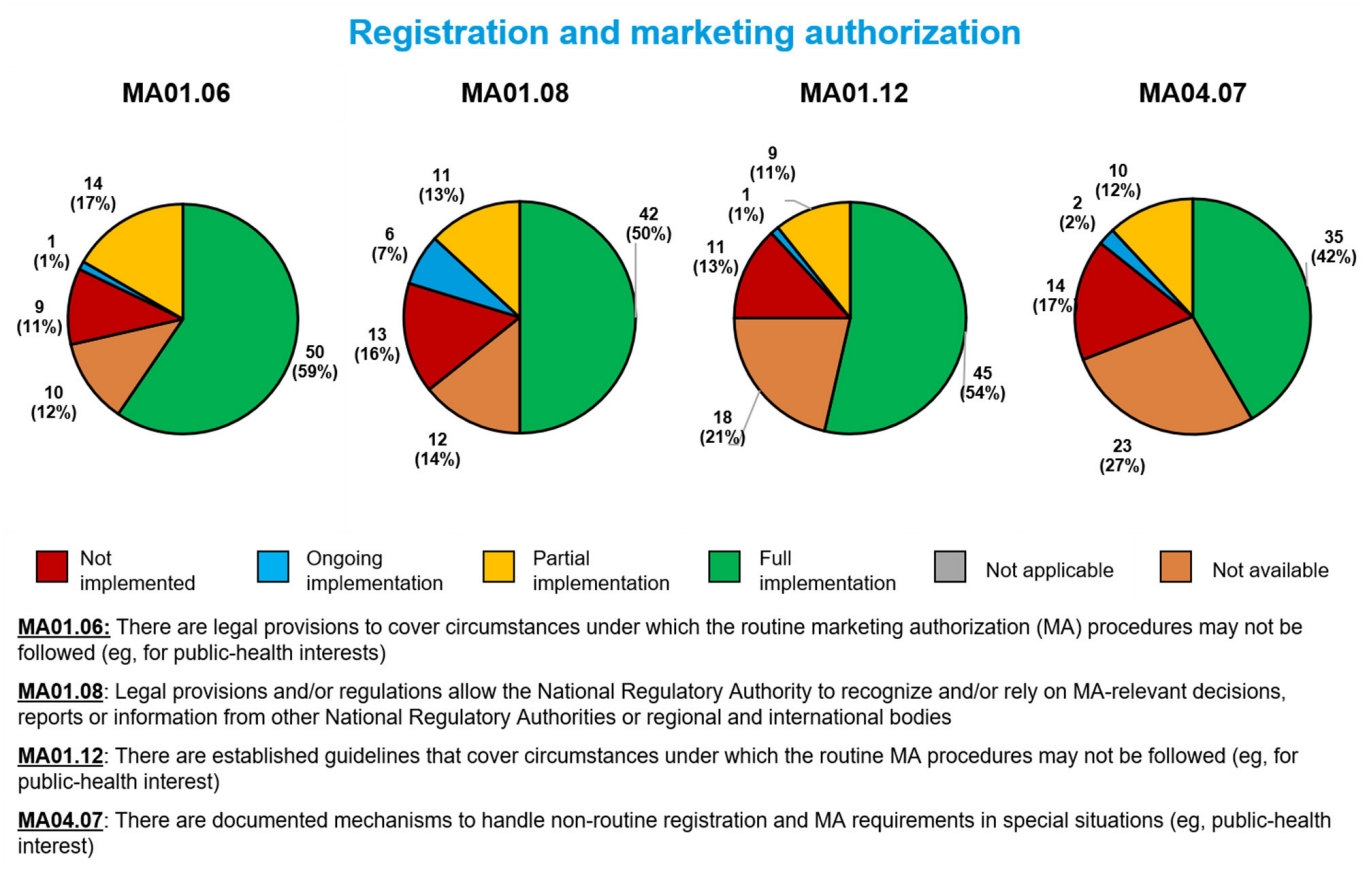
Implementation of four WHO Global Benchmarking Tool sub-indicators related to marketing authorization during a public health emergency, among 84 Member States benchmarked from 2016 to 2020.

For the four sub-indicators related to registration and marketing authorization of medical products during a public health emergency, the most often fully implemented was the legal ability to fast-track marketing authorization applications (MA01.06, 50 Member States, 59%, Figure 3). The least often fully implemented involved documented mechanisms for handling non-routine marketing authorization applications (MA04.07, 35 Member States, 42%). Regarding the remaining sub-indicators, 42 Member States (50%) had fully implemented a sub-indicator related to the use of reliance in marketing authorization decisions (MA01.08), and 45 (54%) had fully implemented a sub-indicator related to guidelines for handling fast-track marketing authorization applications during public health emergencies (MA01.12).

### Pharmacovigilance Sub-indicator

**Figure 4:**
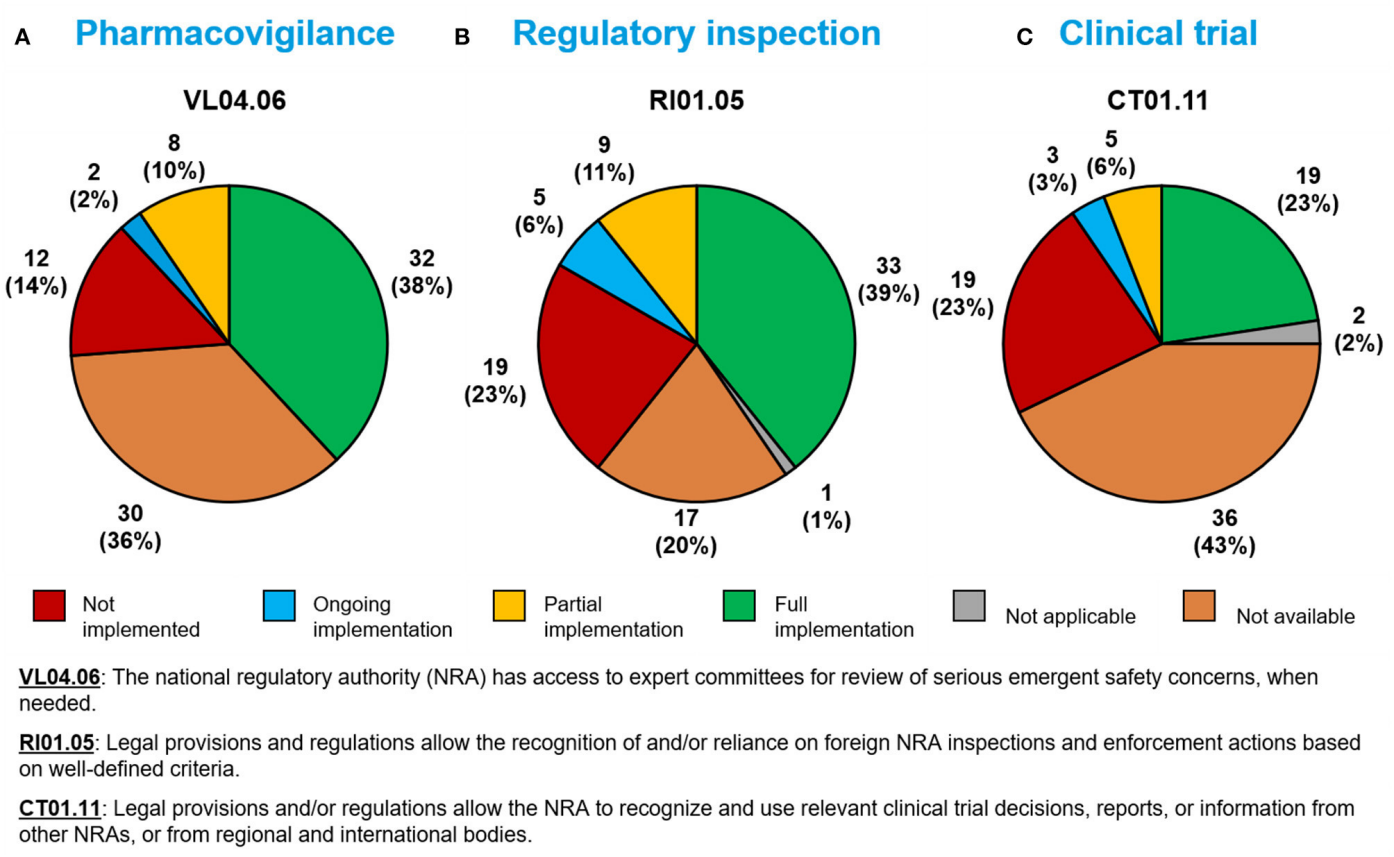
Implementation of WHO Global Benchmarking Tool sub-indicators related to **(A)** pharmacovigilance, **(B)** regulatory inspection, and **(C)** clinical trials during a public health emergency, among 84 Member States benchmarked from 2016–2020.

Thirty-two Member States (38%) had fully implemented a sub-indicator related to possessing expert committees on serious adverse events during a public health emergency (VL04.06; Figure 4).

### Regulatory Inspection Sub-indicator

Thirty-three Member States (39%) had fully implemented a sub-indicator related to the use of reliance in regulatory inspection decisions during a public health emergency (RI01.05; Figure 4).

### Clinical Trial Sub-indicator

Nineteen Member States (23%) had fully implemented a sub-indicator related to the use of reliance in clinical trial decisions (CT01.11; Figure 4).

### Full Implementation Across Sub-indicators

Only 10 Member States (12%) had fully implemented all 10 sub-indicators related to public health emergencies (Table 4), whereas 34 (40%) had fully implemented ≥50% of the indicators. Twenty Member States (24%) had not implemented any of the 10 sub-indicators.

**Table 4:** Full implementation of WHO global benchmarking tool sub-indicators relevant to public health emergencies among 84 member states benchmarked from 2016 to 2020.

**Emergency sub-indicators implementation (%)**	**Member states fully implementing, *n* (%)**
100	10 (12)
≥90	16 (19)
≥80	20 (24)
≥70	24 (29)
≥60	29 (35)
≥50	34 (40)
0	20 (24)

## Discussion

Our results show that regulatory systems in many LMICs are at risk during public health emergencies due to limited preparedness. For the 10 GBT sub-indicators analyzed, full implementation ranged from 23% of Member States (for the use of reliance in clinical trial decisions) to 59% of Member States (for the legal ability to fast-track marketing authorization applications). The majority of Member States displayed fragmented implementation of the various sub-indicators; only 12% had fully implemented all 10. Our results underscore the urgent need to strengthen regulatory systems around the world, so they can properly respond to public health emergencies.

On some occasions, countries may bypass or waive regulatory requirements during a public health emergency, such as the current COVID-19 pandemic or the 2009 influenza pandemic, to avoid the spread of disease. However, this approach is case-based and may not effectively or uniformly provide timely access to safe, quality medical products. It can undermine a country's regulatory system, decrease public trust in its regulators, and lead to the politicization of regulatory decisions. It can also become an excuse for not investing properly in regulatory systems.

Thus, a concerted effort at the country level is needed to address gaps in emergency regulatory procedures. First, all countries should complete a situational gap analysis for regulatory emergency preparedness, the primary tool for which is the GBT. This will enable them to identify where bottlenecks and barriers exist. Common regulatory challenges are both administrative (e.g., slow, rigid, lengthy regulatory pathways; multilayer decision-making processes; limited human, financial, and physical resources) and technical (e.g., competency and knowledge gaps, especially with regard to new, complex products). Second, countries must make provisions for laws, regulations, and guidelines for streamlining and fast-tracking product approval during emergencies, and they should practice using these provisions in simulation exercises, ideally during the inter-pandemic period (14). WHO has made one such simulation exercise, developed for COVID-19, available online (15). Third, in emergencies, countries should rely on the decisions of trusted NRAs (Stringent Regulatory Authorities or future WHO-Listed Authorities) and the WHO Prequalification Program, including the Emergency Use Listing (EUL) (16, 17). [Once the framework is complete, WHO plans to designate NRAs that perform at GBT Maturity Level 3 and 4 as WHO-Listed Authorities, following a performance evaluation process; this should facilitate reliance and trust among Member States (10)]. Countries should recognize that Good Regulatory Practices, regional and international regulatory harmonization, and convergence promote reliance and reduce regulatory hurdles, thus improving timely access to life-saving medical products during public health emergencies (16). Finally, before being faced with an emergency, countries should consider how they can build the trust necessary for regulatory reliance, adjust their legal and regulatory frameworks accordingly, and implement procedures to promote and facilitate cooperation with other NRAs (18).

Importantly, preparing for public health emergencies does not end with creating pathways for swift product approval. In the post-marketing authorization realm, NRAs must regularly update their systems for information sharing and safety monitoring and evaluation. Given the limited data typically available at the time of emergency authorizations for new products, post-marketing surveillance for safety and effectiveness is critically important. NRAs must immediately share reports of suspected lack of effectiveness or adverse reactions with manufacturers, marketing authorization holders, and the global community (16).

Fortunately, abundant evidence from recent decades shows that, despite challenges and resource limitations, regulatory systems in LMICs have improved—and are continuing to gain strength. Since 1997, WHO has worked with more than 150 countries to improve their regulatory systems and documented improvement in more than 100 of them (19). To highlight one example, since 2014, WHO and other partners have systematically worked with 16 LMIC priority countries to enhance their regulatory capacity, as part of WHO's High Level Implementation Plans for Pandemic Influenza Preparedness Partnership Contributions. In the intervening years, 15 of the 16 priority countries have undergone benchmarking and 11 have improved either in maturity level or in the percentage of sub-indicators implemented related to regulatory capacity (20). Two countries have achieved GBT Maturity Level 3. WHO has also supported 32 additional priority countries in enhancing their regulatory pathways for timely approval of pandemic influenza products (20). Improvements in these pathways have been observed in 27 of the targeted countries.

In the next 5 years, WHO's goal is for at least 10 LMICs to have improved their regulatory systems' ability to address public health emergencies, including adopting regulatory provisions for reliance, a fast-track registration process, and an effective pharmacovigilance system (6). WHO helps Member States strengthen their regulatory systems for medical products by setting norms and standards; supporting harmonization; promoting smart regulation; helping countries identify their strengths and gaps, using the GBT; providing specialized technical assistance, networking, and capacity building opportunities; and offering advice (10). Benchmarking, which facilitates WHO's capacity-building efforts, is offered to any country that requests it. Once benchmarking is complete and an IDP is in place, WHO can tailor support to a country's needs, offering learning, twinning, and networking opportunities that target areas for improvement. WHO can also provide specialized technical assistance to NRAs and governments to address regulatory gaps, mainly related to legal frameworks, expedited regulatory pathways, pharmacovigilance, and reliance.

In addition, WHO seeks to assist Member States by releasing guidance on regulatory matters relevant to public health emergencies. For example, it recently released the Good Regulatory Practices Guideline, which describes best practices for developing, implementing, and maintaining regulatory instruments (laws, regulations, guidelines) to achieve public health policy objectives in the most efficient way (17). Another recently released guideline describes Good Reliance Practice, also with the goal of enhancing regulatory efficiency (18). Finally, in May 2017, WHO created an EUL procedure to guide NRAs in expediting the availability of previously unlicensed medical products in public health emergencies. This procedure includes review of a product's safety, performance, and quality, as well as risk management, surveillance, and communication provisions (6).

WHO recognizes that an increasing number of entities are involved in efforts to strengthen regulatory systems at the country, regional, and global level. It also recognizes the value of networks, collaboration, and coordination in supporting Member States to meet targeted objectives and strengthen their regulatory systems, particularly given the limited resources available. It is crucial that various players work together to avoid duplication of effort and to harmonize their approaches and minimize confusion. For this reason, WHO has begun to convene coalitions of interested parties (CIPs). A CIP brings a national government together with donors and partners to coordinate priorities and resources for implementing that country's IDP (11). The CIP approach was pioneered in Bangladesh, and based on the experiences at this pilot site, the WHO is releasing CIP guidance for responding to needs identified using the GBT (21). In this manner, WHO seeks to target resources so that they will have the greatest impact.

This study has several limitations. First, it only includes information from WHO Member States that conducted benchmarking. Second, 69% of evaluations resulted from self-benchmarking, which may be less robust than formal benchmarking. However, even in self-benchmarking, NRAs are asked to submit supporting evidence, such as legal documents, national regulatory guidelines, and internal procedures, and many self-benchmarkings are facilitated by WHO staff. Some of the self-benchmarking data in this study have been verified by WHO staff, other self-benchmarking data have not; non-verified benchmarking may be less accurate. Third, some indicators show high proportion of “not available” data which is attributed to the absence of scoring by the NRA during self-benchmarking. Forth, Member States were benchmarked over a 5-year timeline. It is possible that sub-indicators in some of the countries assessed earlier may now have different scores, because the countries' regulatory systems have improved. However, we do not expect major changes to have taken place. For example, WHO found that only three of its LMIC Member States changed maturity level between 2016 and 2020 (all moved from GBT Maturity Level 2 to Maturity Level 3). Fifth, we recognize that factors besides regulatory procedures (e.g., competency, human and financial resources, independence from a country's Ministry of Health, governance, civil unrest) are also important in determining an NRA's ability to respond to public health emergencies.

In conclusion, this study assessed the current regulatory preparedness of LMICs to respond to public health emergencies and showed that only 12% of participating Member States (10 countries) had fully implemented all 10 relevant GBT sub-indicators. None of the 10 countries which fully implemented all sub-indicators is low income country. On the other side 24% (20 countries) had not fully implemented any of the sub-indicators. Seven out of the 20 countries which had not fully implemented any of the sub-indicators are low income, another seven countries are lower middle income, four countries are upper middle income and two remaining countries are high income countries. The results presented here may be considered a baseline. We hope to complete a follow-up study that looks at how Member States improve in the next 2–3 years, and in particular how well they address gaps identified through benchmarking. We note that several LMICs have already shown that a country does not necessarily need a high income to invest in its regulatory system: Recently, Tanzania, Ghana, and Serbia have attained GBT Maturity Level 3. In short, WHO looks forward to working with Member States and other partners to improve regulatory preparedness for public health emergencies.

## Data Availability Statement

The datasets presented in this article are not readily available because countries that participated in the WHO benchmarkings did not agree to share their data with third parties. Therefore the information they supplied would remain confidential. Requests to access the datasets should be directed to the corresponding author, Alireza Khadem Broojerdi, khadembroojerdia@who.int.

## Author Contributions

AK: conceptualization, study design, literature search, data collection, data analysis and interpretation, visualization, validation, writing the original draft, review, and editing. CA, RO, MR, and HS: literature search, data collection, data analysis and interpretation, visualization, validation, writing the original draft, review, and editing. All authors contributed to the article and approved the submitted version.

## Conflict of Interest

The authors declare that the research was conducted in the absence of any commercial or financial relationships that could be construed as a potential conflict of interest.

## Publisher's Note

All claims expressed in this article are solely those of the authors and do not necessarily represent those of their affiliated organizations, or those of the publisher, the editors and the reviewers. Any product that may be evaluated in this article, or claim that may be made by its manufacturer, is not guaranteed or endorsed by the publisher.

## References

[1] Palkonyay L Fatima H. A decade of adaptation: regulatory contributions of the World Health Organization to the Global Action Plan for Influenza Vaccines (2006-2016). Vaccine. (2016) 34:5414–9. 10.1016/j.vaccine.2016.07.025 27498212

[2] World Health Organization. Main Operational Lessons Learnt from the WHO Pandemic Influenza A (H1N1) Vaccine Deployment Initiative (2011). Available online at: https://apps.who.int/iris/handle/10665/44711 (accessed January 26, 2020).

[3] Decina D Fournier-Caruana J Takane M Ostad Ali Dehaghi R Sutter R. Regulatory aspects of Sabin type 2 withdrawal from trivalent oral poliovirus vaccine: process and lessons learned. J Infect Dis. (2017) 216(Suppl. 1):S46–51. 10.1093/infdis/jiw564 28838164 PMC5853659

[4] Wolf J Bruno S Eichberg M Jannat R Rudo S VanRheenen S. Applying lessons from the Ebola vaccine experience for SARS-CoV-2 and other epidemic pathogens. NPJ Vaccines. (2020) 5:51. 10.1038/s41541-020-0204-7 32566261 PMC7295741

[5] World Health Organization. A67/30. Access to Essential Medicines (2014). Available online at: https://apps.who.int/gb/ebwha/pdf_files/WHA67/A67_30-en.pdf (accessed January 26, 2021).

[6] World Health Organization. Delivering Quality-Assured Medical Products for All: 2019-2023 (2019). Available online at: https://www.who.int/publications/i/item/WHO-MVP-RHT-2019.01 (accessed January 26, 2021).

[7] Preston C Valdez ML Bond K. Strengthening medical product regulation in low- and middle-income countries. PLoS Med. (2012) 9:e1001327. 10.1371/journal.pmed.1001327 23109912 PMC3479087

[8] World Health Organization. Road Map for Access to Medicines, Vaccines and Other Health Products: 2019-2023 (2019). Available online at: https://apps.who.int/iris/handle/10665/330145 (accessed January 26, 2021).

[9] World Health Organization. Thirteenth General Program of Work 2019-2023: Promote Health, Keep the World Safe, Serve the Vulnerable (2019). Available online at: https://www.who.int/about/what-we-do/thirteenth-general-programme-of-work-2019-−2023 (accessed January 26, 2021).

[10] Khadem Broojerdi A Baran Sillo H Ostad Ali Dehaghi R Ward M Refaat M Parry J. The World Health Organization Global Benchmarking Tool: an instrument to strengthen medical products regulation and promote universal health coverage. Front Med. (2020) 7:457. 10.3389/fmed.2020.00457 32974367 PMC7466745

[11] Guzman J O'Connell E Kikule K Hafner T. The WHO Global Benchmarking Tool: a game changer for strengthening national regulatory capacity. BMJ Glob Health. (2020) 5:e003181. 10.1136/bmjgh-2020-003181 32784212 PMC7418656

[12] World Bank. World Bank Open Data. Available online at: https://data.worldbank.org/ (accessed January 26, 2021).

[13] World Bank. World Bank Country and Lending Groups. Available online at: https://datahelpdesk.worldbank.org/knowledgebase/articles/906519-world-bank-country-and-lending-groups (accessed March 2, 2021).

[14] World Health Organization. Guidance on Developing a National Deployment and Vaccination Plan for COVID-19 Vaccines (2020). Available online at: https://www.who.int/publications/i/item/WHO-2019-nCoV-Vaccine_deployment-2020.1 (accessed January 26, 2021).

[15] World Health Organization. Coronavirus Disease (COVID-19) Training: Simulation Exercise. Available online at: https://www.who.int/emergencies/diseases/novel-coronavirus-2019/training (accessed March 2, 2021).

[16] Lumpkin MM Lim JCW. Pandemic best regulatory practices: an urgent need in the COVID-19 pandemic. Clin Pharmacol Ther. (2020) 108:703–5. 10.1002/cpt.1932 32498131 PMC7300901

[17] World Health Organization. Good regulatory practices in the regulation of medical products. WHO Expert Committee on Specifications for Pharmaceutical Preparations: Fifty-fifth report. Technical Report Series 2021; No 1033 (Annex 11). Geneva: World Health Organization (2021).

[18] World Health Organization. Good reliance practices in the regulation of medical products: high level principles and considerations. WHO Expert Committee on Specifications for Pharmaceutical Preparations: Fifty-fifth report. Technical Report Series 2021; No. 1033 (Annex 10). Geneva: World Health Organization (2021).

[19] Belgharbi L Broojerdi A Rodriguez-Hernandez C Wood D. Regulation of vaccines in low- and middle-income countries. In: Plotkin S Orenstein W Offit P Edwards K, eds. Plotkin's Vaccines. 7th ed. Amsterdam: Elsevier (2018). p. 1573–83.

[20] World Health Organization. Regulatory Systems Strengthening Database. Geneva: World Health Organization (2021).

[21] World Health Organization. Terms of Reference for the Coalition of Interested Parties (2021). Available online at: https://www.who.int/publications/m/item/terms-of-reference-for-the-coalition-of-interested-parties (accessed May 27, 2021).

